# Enhanced case finding and self-isolation measures in the early phase of SARS-CoV-2 Omicron transmission, Osaka, Japan, December 2021–January 2022

**DOI:** 10.5365/wpsar.2025.16.2.1129

**Published:** 2025-04-14

**Authors:** Miho Kobayashi, Kensaku Kakimoto, Yuichiro Yahata, Yusuke Kobayashi, Hitomi Nagai, Chisato Tanikake, Kazumi Fukumura, Keiko Date, Hiromi Murata, Sae Kitagawa, Yuki Yoshida, Yui Kamoda, Miho Akazaki, Masaaki Tanabe, Chika Shirai, Tomoe Shimada, Taro Kamigaki, Tsuyoshi Sekizuka, Makoto Kuroda, Tomimasa Sunagawa

**Affiliations:** a Field Epidemiology Training Program, National Institute of Infectious Diseases, Tokyo, Japan.; b Gunma Prefectural Institute of Public Health and Environmental Sciences, Gunma, Japan.; c Osaka Institute of Public Health, Osaka, Japan.; d Center for Field Epidemic Intelligence, Research and Professional Development, National Institute of Infectious Diseases, Tokyo, Japan.; e Center for Surveillance, Immunization and Epidemiologic Research, National Institute of Infectious Diseases, Tokyo, Japan.; f Ibaraki Public Health Center, Osaka, Japan.; g Moriguchi Public Health Center, Osaka, Japan.; h Neyagawa City Public Health Center, Osaka, Japan.; i Hirakata City Public Health Center, Osaka, Japan.; j Center for Pathogen Genomics, National Institute of Infectious Diseases, Tokyo, Japan.

## Abstract

**Objective:**

The severe acute respiratory syndrome coronavirus 2 (SARS-CoV-2) variant B.1.1.529 (Omicron) was first detected in Japan in November 2021. In Osaka, public health centres subsequently increased active case finding and encouraged self-isolation. This study investigated the effectiveness of these countermeasures.

**Methods:**

Cases targeted for analysis were persons who had neither recently travelled abroad nor had contact with foreign tourists but tested positive for SARS-CoV-2 between 24 November 2021 and 4 January 2022 and were suspected or confirmed to have the Omicron variant. We performed a descriptive analysis and calculated the reproduction number (*R*) for each generation using the branching process method. Genomic sequencing data were analysed to plot a haplotype network.

**Results:**

A total of 251 cases were analysed. The median age was 30 years, and 46% (115/251) were in their 20s or younger. The first Omicron case in Osaka was detected on 21 December 2021. Local public health centres conducted health monitoring and contact tracing. We analysed *R*, using information from six clusters, including 42 pairs with a clear relationship between the case and the infected contact (infector–infectee pairs); the clusters had 19, 21 and 2 cases in each subsequent generation. The basic *R* (*t* = 0) was estimated to be 3.2, and subsequent generations (*t* = 1, 2) of *R* decreased to 1.1 and 0.1, respectively. The haplotype network showed that these cases constituted a monophyletic group with others detected around Osaka, indicating that these case-related clusters had been contained and were not involved in the nationwide Omicron waves.

**Discussion:**

Active case finding and self-isolation were found to be effective in limiting the spread of an emerging novel variant.

COVID-19, which is caused by severe acute respiratory syndrome coronavirus 2 (SARS-CoV-2), is an infectious disease first reported as unknown pneumonia in China in December 2019; COVID-19 was declared a pandemic by the World Health Organization (WHO) in March 2020. (*1*, *2*) In Japan, the first domestic patient with COVID-19 was identified in January 2020. (*3*) From that point on, contact tracing was conducted, mainly by public health centres (PHCs). By June 2021, PHCs had interviewed every person suspected to have COVID-19, both prospectively and retrospectively, and recommended self-isolation and SARS-CoV-2 tests to their contacts. From July to September 2021, in Japan there was a sudden surge of COVID-19 cases infected with the Delta variant of SARS-CoV-2, which led many local governments to change their policies on epidemiological investigations. (*4*, *5*) After that, contact tracing focused mainly on high-risk groups, such as those attending day care facilities for elderly people and inpatients at hospitals. (*6*)

In November 2021, a novel variant of SARS-CoV-2, B.1.1.529, was reported in South Africa. WHO named this variant Omicron and subsequently classified it as a variant of concern. (*7*) In Japan, the Omicron variant was first detected in November 2021 in a patient who had travelled overseas. Subsequently, in December 2021, a case who had no history of overseas travel was identified. At that time, the Japanese government had implemented stringent border controls, so only a few foreign nationals and Japanese citizens could travel overseas for business. (*8*) Because of insufficient information about the transmissibility and severity of the Omicron variant, the Japanese Ministry of Health, Labour and Welfare instructed local governments to expand the target population for contact tracing. (*9*)

PHCs in Osaka Prefecture, the second most densely populated prefecture in western Japan, in the Kansai Region, began enhanced active case finding and encouraged self-isolation of cases and contacts during the early phase of the Omicron wave. However, elsewhere in Japan (i.e. outside the Kansai Region), measures focused mainly on high-risk groups because they were implemented before the emergence of the Omicron variant. This study aimed to assess the effectiveness of these countermeasures by calculating the reproduction number (*R*) for COVID-19 and performing genomic analysis.

## Methods

Cases targeted for inclusion in the analysis were persons who had neither recently travelled abroad nor had contact with foreign tourists but who had tested positive for SARS-CoV-2 between 24 November 2021 and 4 January 2022 and were L452R mutation–negative by reverse transcription–polymerase chain reaction (RT–PCR) or confirmed to have the Omicron variant by whole-genome sequencing analysis. (*10*, *11*) PHCs classified contacts of each case using the following categories: (1) close contact, (2) other contact and (3) no direct contact with cases (**Table 1**). As part of the contact tracing procedure, PHCs requested that close contacts and other contacts take a SARS-CoV-2 test, and those who tested negative were monitored to see if they developed any symptoms. Those who developed symptoms were re-tested.

**Table 1:** Follow up with and definitions of contacts exposed to the Omicron variant of SARS-CoV-2, Osaka, Japan, December 2021–January 2022

Description and follow up	Category		
	Close contact	Other contact	No direct contact
**Definition**	**Face-to-face contact within 1 m of case and for at least 15 minutes**	**Frequent contact with case even if physical distance was maintained** **Living with case in dormitory or another place with shared facilities, such as dining area or bathrooms**	**Attends the same facility, workplace, or school as the case**
**Health monitoring**	**Yes**	**Yes**	**Yes**
**Testing**	**Yes**	**Yes**	**Yes**
**Self-isolation request**	**Stay at home or at designated accommodation provided by the local government**	**Refrain from going outside**	**Avoid contact with other people**

PHCs in Osaka treated the following persons as contacts requiring self-isolation: (1) a person who had face-to-face contact with a confirmed case within 1 m and for at least 15 minutes; (2) a person who had frequent contact with a confirmed case, even if the proper physical distance was maintained; and (3) a person in a living situation with a confirmed case in which there were communal spaces, such as a dormitory with shared dining areas and bathrooms. Contacts were asked to self-isolate in a local government-designated facility or at home, and their health was monitored until 14 days after their last exposure. In collaboration with local PHCs, we estimated the source of infection and established epidemiological links based on the outbreak investigation data collected by PHCs through 4 January 2022.

The *R* for each generation was calculated using the branching process method. (*12*) We selected events in which there was a clear relationship between the case and the infected contact (infector–infectee pairs), estimated from the date of onset in pairs. Specifically, we selected pairs with distinct onset dates from among the six clusters for which data were available to collect information about epidemiological links. The case with the earlier onset date was defined as the infector and the case with the later onset date as the infectee. To calculate the *R* for each generation, we collected 42 infector–infectee pairs from the six clusters.

Haplotype network analysis is used to describe ancestral relationships between genomic data sets. We conducted this analysis using genomic sequencing data from the National Institute of Infectious Diseases and from local public health institutes to explore the introduction and spread of the Omicron variant. (*13*, *14*)

## Results

A total of 251 cases detected through PHC investigations were included in the analysis. The characteristics of these cases are shown in **Table 2**. The median age was 30 years (interquartile range, 17–49 years), and 46% (115/251) were in their 20s or younger. Altogether, 70% (175/251) were symptomatic, and 57% (142/251) had been vaccinated twice. People who did not have direct contact with cases did not test positive for SARS-CoV-2.

**Table 2:** Demographic characteristics of cases^a^ infected with the Omicron variant of SARS-CoV-2, Osaka, Japan, December 2021–January 2022 (*n* = 251)

Characteristic	No.	%
**Sex**		
**Male**	**119**	**47**
**Female**	**115**	**46**
**Unknown**	**17**	**7**
**Age group (years)**		
** < 10**	**23**	**9**
**10–19**	**47**	**19**
**20–29**	**45**	**18**
**30–39**	**32**	**13**
**40–49**	**30**	**12**
**50–59**	**24**	**10**
**60–69**	**10**	**4**
**70–79**	**23**	**9**
**Unknown**	**17**	**7**
**Clinical manifestationsb**		
**Symptomatic**	**175**	**70**
**Asymptomatic**	**29**	**12**
**Unknown**	**47**	**19**
**Deathsb**	**0**	**0**
**Vaccination historyc**		
**Two vaccinations**	**142**	**57**
**One vaccination**	**5**	**2**
**Unvaccinated**	**29**	**12**
**Not eligible for vaccinationd**	**22**	**9**
**Unknown**	**53**	**21**
**Testing for variant**		
**Whole-genome sequencing**	**114**	**45**
**Only L452R–negative**	**137**	**55**

^a^ Case definition; persons who had neither recently travelled abroad nor had contact with foreign tourists but who had tested positive for SARS-CoV-2 between  24 November 2021 and 4 January 2022 and were L452R mutation–negative by reverse transcription–polymerase chain reaction (RT–PCR) or confirmed to have the Omicron variant by whole-genome sequencing analysis.

^b^ At the time of diagnosis.

^c^ Booster doses started in Japan on 1 December 2021.

^d^ As of 4 January 2022, children younger than 12 years had not been approved for the COVID-19 vaccine.

**Fig. 1:**
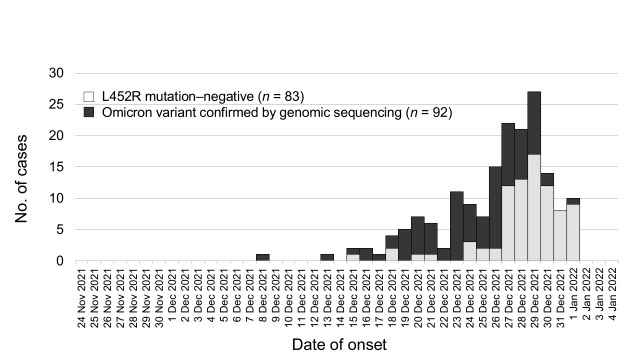
Epidemic curve of COVID-19 in Osaka, Japan, 24 November 2021 to 4 January 2022 (n = 175)

The epidemic curve by date of onset is shown in **Fig. 1**. The first case infected with the Omicron variant in Osaka had an onset date of 8 December 2021; the Omicron variant was detected on 18 December; and the positive test was confirmed using whole-genome sequencing on 21 December.

[**insert** Figure 1]

Among the 251 cases, we identified eight clusters based on the epidemiological information collected by the PHCs, and the data from six clusters were included in the branching process calculation of *R*. Two of the eight clusters were excluded from the analysis because there were no further generations. For two clusters, the contact occurred at home; the remainder of the contacts occurred at a dinner party, school, an after-school setting, and a day care facility for elderly people. Immediately after the index case was detected by a PHC, the facility associated with a COVID-19 cluster was temporarily closed. Close contacts included those who lived with the case and those who had had contact without wearing a mask. Close contacts were required to self-isolate for 14 days in their homes or at accommodation designated by the local public health authority. People who worked or stayed with colleagues, classmates or facility staff who had COVID-19 were classified as other contacts. At the time of contact, most people had been wearing masks. The PHCs asked contacts to refrain from going out. Those who had had no direct contact with a case, such as students in different classrooms at a school, were eligible for health monitoring and testing. They were not asked to observe substantial behavioural restrictions, but the PHCs urged them to avoid contact with persons other than classmates and family members. The health condition of all persons in each cluster was monitored and tested until 14 days after their last contact with a case.

**Fig. 2:**
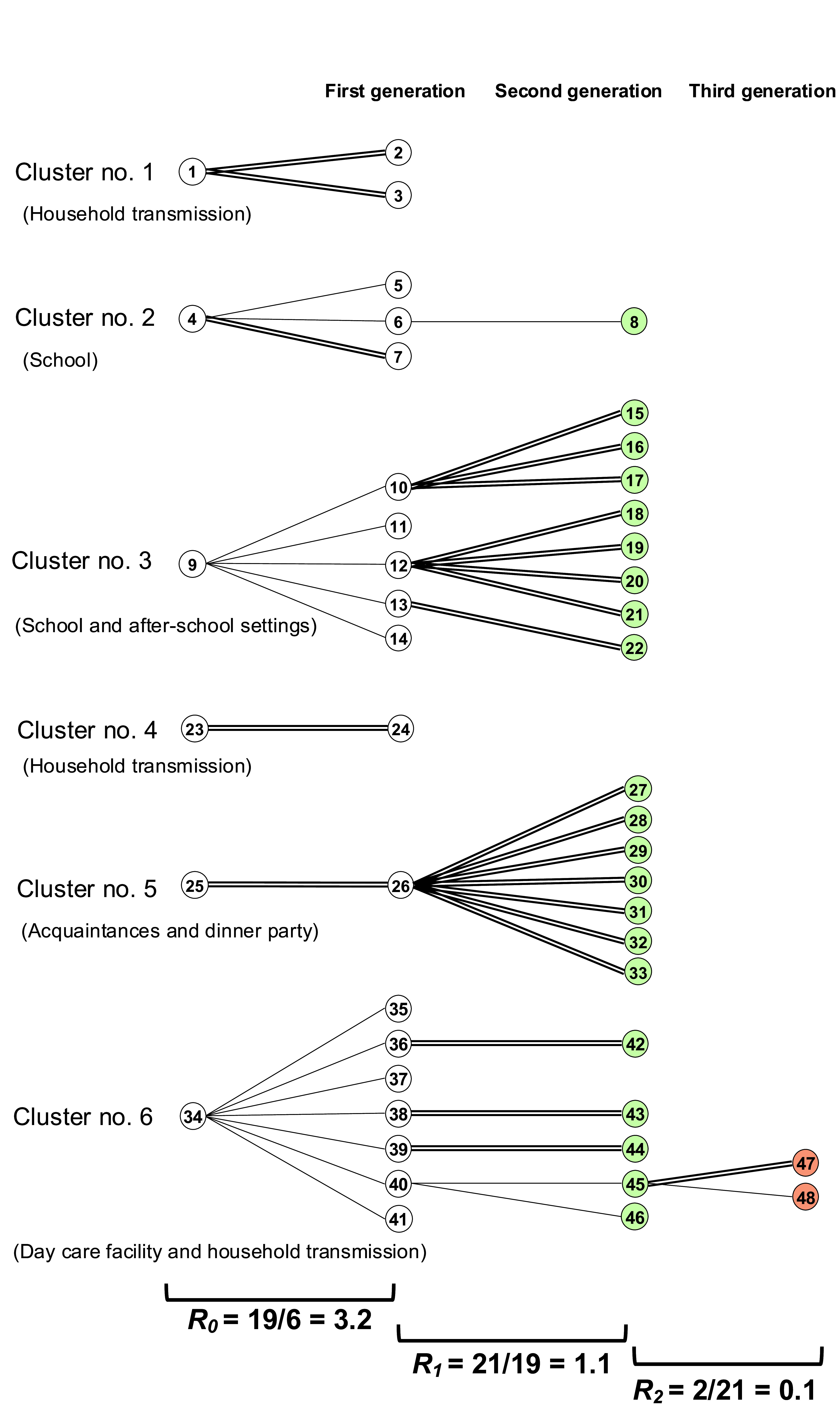
Linkage between COVID-19 cases and infected contacts (infector–infectee pairs) and reproduction number (R), Osaka, Japan, 24 November 2021 to 4 January 2022

**Fig. 2** shows the relationship between infector–infectee pairs in each generation. This tree includes information about the six analysed clusters (originating from the six index cases), which included 19 cases in the first generation, 21 cases in the second and 2 cases in the third. Thus, a total of 48 cases detected through PHC investigations were included in this analysis.

Cluster 1 involved household transmission. Using active case finding, PHCs included family members living together and relatives living apart as close contacts. Testing and health monitoring were performed for the colleagues of the index case (no. 1) and all attendees and staff at the nursery school that the first generation cases (nos. 2 and 3) attended. As a result of the epidemiological investigation conducted by the PHCs, it was determined that no further transmission occurred among the contacts of the cases, including within the family, and at the school and the kindergarten.

Clusters 2 and 3 originated, respectively, from school and after-school settings. Although the two schools are located in the same city, we could not identify any relationship between the two outbreaks. Each pair had contact in the classroom, in the after-school setting or at home.

[**insert** Figure 2]

Cluster 4 involved household transmission. The PHC investigated the work contacts of case 23, but no additional cases were identified.

In cluster 5, cases 25 and 26 were acquaintances who frequently met and had meals together. All second generation cases (nos. 27–33) were suspected to have become infected when they had meals with case 26.

Cluster 6 involved transmission at a day care facility for elderly people. The first case (no. 34) was a staff member. After the case was identified, people who attended the facility and other staff were also diagnosed with SARS-CoV-2 (nos. 35–41). While providing rehabilitation services at the facility, staff wore masks and kept a distance of at least 1.5 m from each other; attendees also followed these precautions. However, the windows were not open to provide ventilation due to safety concerns. The facility was temporarily closed for 14 days. Three cases (nos. 42–44) involved transmissions from attendees to household members. Case 47 also involved household transmission; case 45 was suspected to have been infected by case 40 at another rehabilitation facility. Cases 40 and 46 were acquaintances. Case 48 was a relative of case 45, who lived separately but had contact with case 45; case 45 infected case 48.

In the second generation, the calculated *R* for cases reached 1.1, and in the third generation, it dropped to 0.1. Contacts in the first and second generations were identified by PHCs and were placed under health monitoring. All the primary cases in each pair developed symptoms that led to suspicion of SARS-CoV-2 infection.

**Fig. 3:**
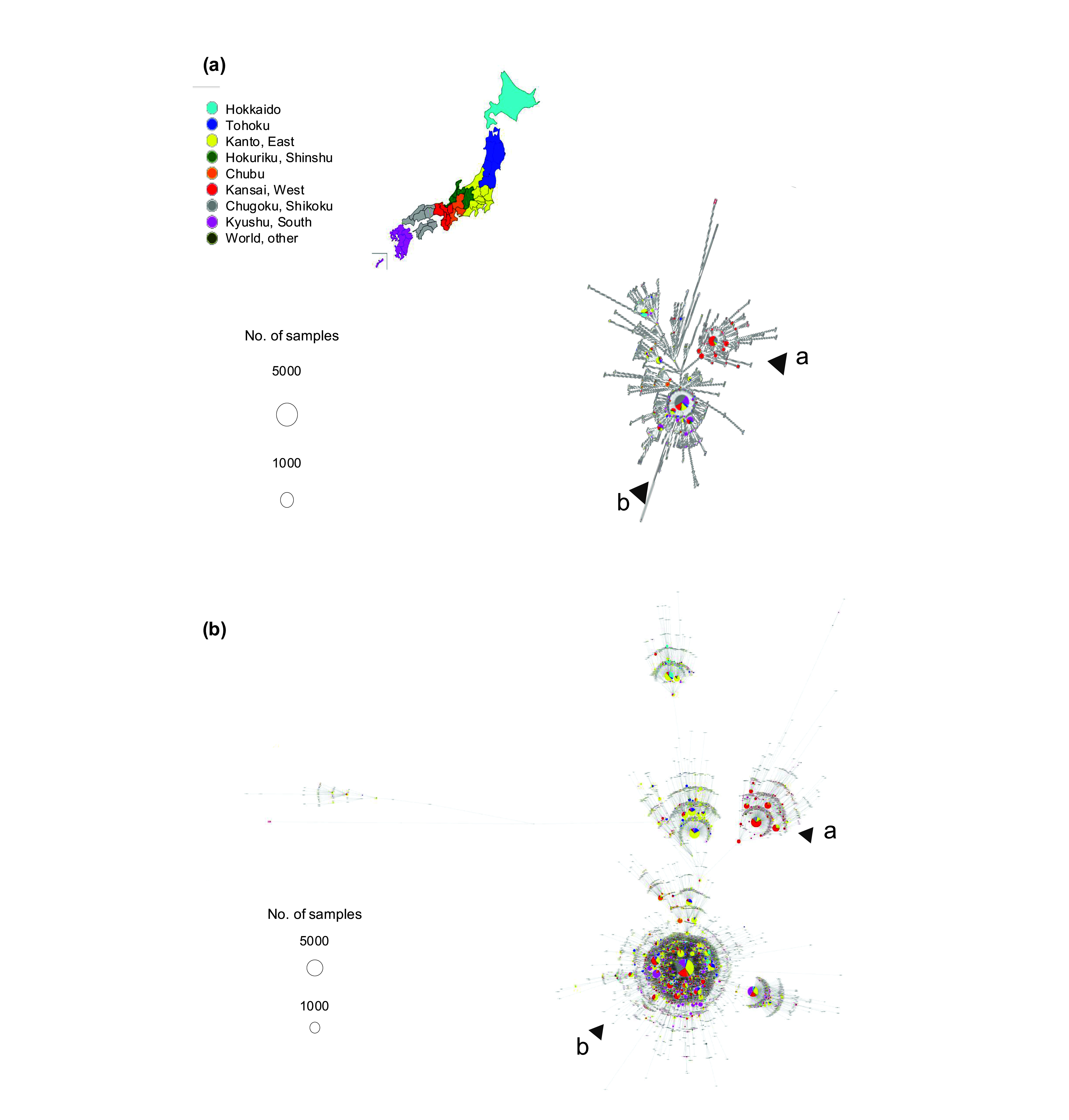
Haplotype network of the Omicron variant detected in Japan (a) as of 14 January 2022 and (b) as of 18 February 2022

**Fig. 3** describes the haplotype networks in which the distribution in each node comprises the cases and shows the identical genome sequence of SARS-CoV-2 between 14 January and 18 February 2022 in Japan. The haplotype network revealed that these cases constituted a monophyletic group, with others detected in Osaka and the surrounding area (i.e. the Kansai Region). The type circulating in the Kansai Region is designated as node a, and its range did not expand much between January (**Fig. 3a**) and February (**Fig. 3b**) of 2022. However, as also shown in **Fig. 3**, node b expanded from the Kyushu, South Region and the Chugoku, Shikoku Region to other regions. This means that other types detected in other regions, including in Okinawa Prefecture (in the Kyushu, South Region) and the Chugoku, Shikoku Region, expanded nationwide.

[**insert** Figure 3]

## Discussion

We described the epidemiology of the early phase of the Omicron wave in Osaka Prefecture, Japan, during which PHCs in Osaka increased active case finding and expanded the target population for self-isolation. To evaluate these measures, we calculated the *R* for each generation and found that it had decreased by the second generation. COVID-19 was most often spread in infector–infectee pairs through household transmission. Cases reproduced largely in clusters where people ate and drank together. (*15*) Our data showed the only clusters associated with a day care facility for elderly people reproduced to a third generation. In addition, according to the haplotype network, the Omicron variant detected in the Kansai Region, which includes Osaka Prefecture, showed limited expansion.

Several studies reported that contact tracing and self-isolation in countries and areas had delayed community transmission during the early phase of the COVID-19 pandemic. (*16*-*19*) For example, in United Kingdom of Great Britain and Northern Ireland, from January to March 2020, when case numbers were low, contact tracing likely had a significant impact on the course of the epidemic. (*16*) However, as transmissibility increased, the periods between case detection, contact tracing and self-isolation of contacts became shorter than they had been when the Delta variant emerged and became dominant. For the Omicron variant, isolation after symptom onset was less effective at preventing transmission because of the shortened generation interval. (*20*) Therefore, many countries reached the limit of contact tracing as the number of cases increased. In Japan, from the middle of the Delta wave, contact tracing focused on those at risk of severe disease in periods and regions where the number of COVID-19 cases was particularly high. Based on uncertainty about the emerging Omicron variant, PHCs in Osaka changed the targets for epidemiological investigation in accordance with the national policy at that time. Every PHC in Osaka enhanced control measures and not only requested that contacts self-isolate but also tested a more comprehensive target population of close contacts. As mentioned above, while PHCs considered the details of the outbreak investigation, they also tested those who did not have direct contact with cases. In addition, local governments provided designated accommodation to ensure that infected cases and close contacts complied with self-isolation, and PHCs confirmed the health condition of close contacts via phone. Furthermore, each PHC urged cases to avoid contact with others to prevent the spread of infection. In fact, no one who did not have direct contact with a case tested positive for SARS-CoV-2 in Osaka. Our data suggest that these enhanced measures contributed to mitigating the spread of the emerging Omicron variant in the Kansai Region.

Just after the Omicron variant was first detected in Japan, airport quarantine procedures were strengthened nationwide, and isolation and health monitoring were required for passengers who had been on a flight with a person infected with the Omicron variant. However, local governments responded in different ways to community-acquired cases of infection with the Omicron variant. First, contact tracing had targeted high-risk groups since the Delta wave, and in some areas, contact tracing continued beyond November 2022. The PHCs, hospitals and clinics in Osaka were able to immediately allocate sufficient personnel to perform contact tracing, health monitoring, testing and genomic surveillance because human resources had been adequately maintained. Second, the Omicron variant spread quickly in Okinawa Prefecture and the Chugoku Region from imported cases. (*21*, *22*) Some infected individuals did not respond to requests to participate in epidemiological investigations in these areas for personal or cultural reasons, regardless of whether they could communicate in Japanese. Meanwhile, fewer Omicron variant cases spread to Osaka compared with Okinawa Prefecture and the Chugoku Region. (*21*, *22*) When PHCs in Osaka asked persons who did not meet the definition of a close contact to refrain from going out as much as possible, they were generally cooperative, even though self-isolation was not obligatory. The differences in response may have affected the speed of transmission in each region. The weekly number of new COVID-19 cases per 100 000 population as of 5 January 2022 was higher in Okinawa Prefecture (80.07) and the Chugoku Region (10.46) than in the Kansai Region (6.43), which includes Osaka Prefecture. (*23*) However, by 1 February 2022, the number of new COVID-19 cases in the Kansai Region had increased to 619.15/100 000 population, which was higher than in both Okinawa Prefecture and the Chugoku Region. (*24*) Moreover, as shown in **Fig. 3**, node b of the haplotype network expanded not only in Okinawa Prefecture and the Chugoku Region but also in the Kansai and Kanto Regions. Node a expanded less than node b; thus, node b showed the expansion of the Omicron variant overall, while node a showed a small expansion nationwide. Our data suggest that the early phase of control measures successfully mitigated the spread of the Omicron variant in Osaka.

Our investigation has several limitations. First, the data are based on information available as of 4 January 2022, which might have underestimated the number of cases due to the time lag between diagnosis and reporting. Second, all primary cases in the study were identified in medical facilities or PHCs. We believe that the PHCs diligently performed contact tracing to identify potential contacts; however, persons with mild cases of COVID-19 might not visit a physician, which would lead to some cases being missed, particularly in the primary generation of branches. Therefore, the value of *R* might have been underestimated based on the available data. Third, some of the cases identified as negative by the L452R screening might have been misclassified as Omicron, given that genomic testing was not available for all cases. Fourth, we might not have obtained sufficiently accurate information about the epidemiological links due to recall bias or social desirability bias.

In conclusion, enhanced active case finding and self-isolation for both cases and contacts may have mitigated community transmission in Osaka during the early phase of spread of the emerging Omicron variant. An emerging novel variant has the potential to spread and cause a pandemic. In reality, several sublineages of SARS-CoV-2 detected outside of Osaka were responsible for the nationwide Omicron wave. During the early phase, there was no information about the characteristics of the emerging novel variant, including its transmissibility and severity. PHCs need sufficient time to consider what kind of response is necessary. The enhanced interventions by PHCs in Osaka were effective in delaying the spread during the early phase of infections with a novel variant of COVID-19, suggesting actions that should be considered in the event of a future outbreak of a novel infectious disease.
